# The autism-associated gene chromodomain helicase DNA-binding protein 8 (*CHD8*) regulates noncoding RNAs and autism-related genes

**DOI:** 10.1038/tp.2015.62

**Published:** 2015-05-19

**Authors:** B Wilkinson, N Grepo, B L Thompson, J Kim, K Wang, O V Evgrafov, W Lu, J A Knowles, D B Campbell

**Affiliations:** 1 Development, Stem Cells, and Regeneration Ph.D. Program, University of Southern California, Los Angeles, CA, USA; 2 Zilkha Neurogenetic Institute, Keck School of Medicine, University of Southern California, Los Angeles, CA, USA; 3 Division of Occupational Science and Occupational Therapy, Herman Ostrow School of Dentistry, University of Southern California, Los Angeles, CA, USA; 4 Department of Psychiatry and the Behavioral Sciences, Keck School of Medicine, University of Southern California, Los Angeles, CA, USA; 5 Broad Center for Regenerative Medicine and Stem Cell Research, Keck School of Medicine, University of Southern California, Los Angeles, CA, USA; 6 Department of Biochemistry and Molecular Biology, Keck School of Medicine, University of Southern California, Los Angeles, CA, USA

## Abstract

Chromodomain helicase DNA-binding protein 8 (*CHD8*) was identified as a leading autism spectrum disorder (ASD) candidate gene by whole-exome sequencing and subsequent targeted-sequencing studies. *De novo* loss-of-function mutations were identified in 12 individuals with ASD and zero controls, accounting for a highly significant association. Small interfering RNA-mediated knockdown of *CHD8* in human neural progenitor cells followed by RNA sequencing revealed that CHD8 insufficiency results in altered expression of 1715  genes, including both protein-coding and noncoding RNAs. Among the 10 most changed transcripts, 4 (40%) were noncoding RNAs. The transcriptional changes among protein-coding genes involved a highly interconnected network of genes that are enriched in neuronal development and in previously identified ASD candidate genes. These results suggest that CHD8 insufficiency may be a central hub in neuronal development and ASD risk.

## Introduction

Autism spectrum disorder (ASD) is a complex neurodevelopmental disorder characterized by impairments in social interaction, communication and behavioral flexibility.^1^ Due to the vast clinical and genetic heterogeneity of ASD, the identification of causal genetic determinants has proven challenging.^2, 3, 4^ However, multiple independent studies have now provided substantial evidence for the contribution of *de novo* loss-of-function (LoF) mutations in chromodomain helicase DNA-binding protein 8 (*CHD8*) to ASD.^5, 6, 7, 8, 9^ Initially, exome sequencing on 209 ASD probands and their unaffected family members discovered two *de novo* LoF mutations in *CHD8*.^6^ Subsequent targeted sequencing of 44 candidate genes in 2446 ASD probands identified an additional seven *de novo* LoF mutations in *CHD8*, establishing a frequency of mutation that is the highest of all genes screened.^7^ Furthermore, a second targeted-sequencing study of an additional 3370 children with ASD or developmental delay identified three *de novo* LoF mutations in *CHD8*.^8^ Altogether, these highly significant findings have placed *CHD8* as a genuine ASD risk factor and account for 0.2% (12/6,176) of ASD cases. The *CHD8* LoF mutations have been found throughout the coding region of the gene, with truncating mutations as early as amino acid 62 of the 2581 amino acid CHD8 protein. Truncating mutations were found in the chromodomain, the dex domain and the helicase domain. A detailed map of all the identified *CHD8* LoF mutations was published recently.^9^ In addition to *de novo* mutations in *CHD8*, six potentially disrupting mutations with unknown or inherited modes of transmission have also been identified^8, 9^ along with a balanced chromosomal abnormality leading to disruption of *CHD8*.^10^ *CHD8* LoF mutations have not been found in any of the 8792 controls included in these analyses, emphasizing the impact of *CHD8* LoF mutations on ASD risk.^9^ Phenotypic characterization of individuals with *CHD8* disrupting mutations indicate a subset of ASD that includes macrocephaly, distinct facial features and gastrointestinal difficulties.^8^

Although a critical role of CHD8 in development is revealed by the embryonic lethality of *Chd8* knockout mice,^11^ the function of CHD8 in neural cell lineages has been largely unexplored. As CHD8 actively associates with core transcriptional machinery,^12^ transcription factors^13, 14^ and histone-modifying complexes,^15^ transcriptional dysregulation conferred by CHD8 insufficiency may provide evidence for the neurodevelopmental phenotypes observed in ASD. To emulate the potential effects of the identified LoF mutations, we performed small interfering RNA (siRNA)-mediated knockdown of *CHD8* followed by genome-wide transcriptional profiling through RNA sequencing (RNA-seq). Here, we show that knockdown of *CHD8* in SK-N-SH human neural progenitor cells results in altered expression of a highly interconnected network of genes, which are enriched in several processes essential for neuronal development. Remarkably, numerous previously identified ASD candidate genes are also differentially expressed in response to knockdown of *CHD8*, suggesting a convergence of multiple genes contributing to ASD onset. The data presented here point toward a role of *CHD8* in maintaining the active transcription of neural-specific genes and begins to elucidate the potential contributions of decreased functional CHD8 to the pathogenesis of ASD.

## Materials and methods

### Cell culture

To measure gene expression in human neural progenitor cells, SK-N-SH cells (American Type Culture Collection; Manassas, VA, USA) were maintained in minimal essential medium supplemented with 10% heat-inactivated fetal bovine serum, 1% penicillin/streptomycin, non-essential amino acids, and 1.5 g l^−1^ sodium bicarbonate in 183-cm flasks at 37 °C and 5% CO_2_.

### siRNA transfection

To determine the impact of CHD8 knockdown on gene expression in human neural progenitor cells, SK-N-SH cells were seeded into six-well 10-cm plates and grown for 24 h (~70% confluency) before transfection. Transfections were carried out with either siRNA silencer select negative control No. 1 (catalog no. 4390843, Ambion/Life Technologies; Carlsbad, CA, USA) or siRNA targeting *CHD8* (catalog no. 33582, Ambion/Life Technologies)^16^ at a concentration of 20 nM using Lipofectamine RNAiMAX Reagent (Invitrogen/Life Technologies; Carlsbad, CA, USA) according to the manufacturer's protocol. Cells were then collected 72 h post siRNA transfection and processed for downstream applications. Experiments were performed in quadruplicate.

### Western blot analyses

To determine the degree to which siRNA knockdown of *CHD8* transcript results in decreased CHD8 protein, total protein was isolated using the Illustra triplePrep kit (GE Healthcare; Waukesha, WI, USA) and protein concentration was determined using the DC protein assay (Bio-Rad; Hercules, CA, USA). Total protein (10 μg) was then separated on a 4–20% gradient criterion TGX gel (Bio-Rad) and transferred to a nitrocellulose membrane by capillary transfer at 80 V for 3 h using a Bio-Rad Criterion blotter system. Blots were incubated overnight at 4 °C with anti-CHD8 (catalog no. 7656, Cell Signaling Technology; Danvers, MA, USA) and anti-GAPDH (catalog no. 1228, Cell Signaling Technology) primary antibodies diluted 1:1000 in blocking buffer. Anti-rabbit immunoglobulin G, horseradish-peroxidase-linked secondary antibody (catalog no. 7074, Cell Signaling Technology) was diluted 1:2000 and incubated with the membrane for 1.5 h at room temperature. Signal was developed using SuperSignal West Dura Extended Duration Substrate (Thermo Fisher Scientific; Waltham, MA, USA) and captured on a BioSpectrum Imaging System (UVP; Upland, CA, USA). The total density ratio of CHD8 to GAPDH was determined using the VisionWorks LS version 6.8 software (UVP) and analyzed by Student's paired *t*-test for comparison (*n*=4).

### RNA-seq

To determine genes that are differentially expressed in response to knockdown of *CHD8* on a genome-wide scale, cells were homogenized with QIAshredder spin columns (Qiagen; Valencia, CA, USA) followed by total cellular RNA extraction using the Qiagen RNeasy kit according to the manufacturer's protocol. RNeasy extraction typically excludes fragments <200 bp. Therefore, the data presented here focuses on long noncoding RNAs (ncRNAs). Quality (RNA integrity number) was assessed using an Agilent Technologies 2200 TapeStation Instrument (Agilent Technologies; Santa Clara, CA, USA) and 260/280 absorbance ratios were obtained using the NanoDrop ND-1000 Spectrophotometer (Thermo Fisher Scientific). cDNA library preparation was carried out using the Illumina Truseq Stranded Total RNA Sample Preparation kit and sequencing was performed on the HiSeq2000 sequencer to generate 101 bp single-end reads (Illumina; San Diego, CA, USA). An approximate average of 45 million reads per replicate was obtained for samples treated with siRNA targeting *CHD8* and an average of 40 million reads per replicate was obtained for samples treated with negative control siRNA (Supplementary Table 1).

### RNA-seq analysis

Quality control pre-processing of raw reads was carried out using Cutadapt.^17^ Reads were first mapped to the transcriptome of either UCSC Hg19 or ENSEMBL GrCH37 release 75 and the remaining reads were then mapped to the unannotated regions of the respective human genome build using Tophat2.^18^ The percentage of reads that were aligned to the genome for each replicate along with their respective quality control metrics are outlined in Supplementary Table 1. Following alignment, differential gene expression analysis was carried out using Cuffdiff^19^ along with a supplied GTF file of the corresponding transcriptome to be analyzed. Cuffdiff analysis was performed with default settings with the exception of the masking of all annotated ribosomal RNAs and the no-effective-length-correction option, which was used to reduce overestimation of short transcript expression. Genes with a false discovery rate (*Q*-value) of <0.05 were considered significant. All gene ontology (GO) and protein–protein interaction analyses were carried out using the list of significantly differentially expressed genes identified using the UCSC Hg19 human genome annotation. To explore changes in genes transcribing ncRNAs, we also carried out the RNA-seq analysis using the ENSEMBL GrCH37, release 75, gene annotation, which contains a much larger quantity of ncRNA transcripts compared with the UCSC Hg19 annotated transcriptome.

### Quantitative PCR

To measure the degree to which siRNA knockdown of *CHD8* results in decreased transcript levels and verify the quality of RNA-seq data analysis, cell pellets were processed for RNA isolation using the Qiagen RNeasy kit (Qiagen). Superscipt III (Life Technologies; Grand Island, NY, USA) was used for reverse transcription of cDNA libraries. Quantitative PCR (qPCR) was performed using Life Technologies Taqman Gene Expression qPCR kits on a Life Technologies OneStepPlus real-time PCR machine. Each sample was run in triplicate along with the housekeeping gene, *GAPDH*. Assays used for validation (Life Technologies) include *ABCA1* (Hs01059118_m1), *CHD8* (Hs00394229_m1), *BDNF* (Hs02718934_s1), *CUL1* (Hs01117001_m1), *GAPDH* (Hs9999905_m1), *GJA1* (Hs00748445_s1), *HMGA2* (Hs00171569_m1), *HSPB3* (Hs00937412_s1), *IGF2* (Hs04188276_m1), *IGFBP3* (Hs00365742_g1), *IL1R1* (Hs00991002_m1), *IL8* (Hs00174103_m1), *LINC00473* (Hs00293257_m1), *LINC01021* (Hs01388536_m1), *MMP3* (Hs00968305_m1), *PITX3* (Hs01013935_g1), *SCN2A* (Hs01109877_m1), *SERPINE1* (Hs01126606_m1), *SEMA3D* (Hs00380877_m1), *SYTL2* (Hs00909223_m1), *TGM2* (Hs00190278_m1) and *TNS3* (Hs00994933_m1). Relative quantity of the assayed transcript was determined by the 2^−ΔΔCt^ method using *GAPDH* as a reference.^20^ For RNA-seq validation, the observed qPCR changes were compared with those obtained in RNA-seq data analysis through the use of Spearman and Pearson correlation tests carried out in the R statistical environment using the (cor.test) package.

### Functional annotation and network construction

To begin to functionally characterize and cluster the differentially expressed transcripts, the Database for Annotation, Visualization and Integrated Discovery (DAVID)^21^ was used to analyze for the enrichment of GO terms and UP_TISSUE (Uniprot tissue annotation database) categories. All analyses were carried out with the default options. Terms that were redundant or had a corrected *P-*value >0.05 were eliminated. Disease Association Protein–Protein Link Evaluator^22^ was used to determine direct interactions and connectivity among the protein products of the differentially expressed genes using the default settings. Network construction of the resulting interactions was carried out using Cytoscape version 3.1.1 software.^23^

### ASD candidate genes statistical analysis

To analyze whether or not a lack of functional *CHD8* results in the differential expression of other ASD candidate genes, a list of ASD candidate genes was obtained from the Simons Foundation Autism Research Initiative (SFARI) AutDB database.^24^ A restricted list of ASD candidate genes was then curated by using only those genes with SFARI gene rankings 1–4 (strong evidence–minimal evidence) and S (syndromatic) as previously reported.^25^ Genes above the threshold of reliable detection in Cuffdiff (a total of 13 020 genes) served as the background for enrichment tests, which were measured by the two-tailed Fisher's exact test using the (fisher.test) package in R.

## Results

### Quantitative modeling of *CHD8 de novo* loss-of-function mutations

**Figure 1:**
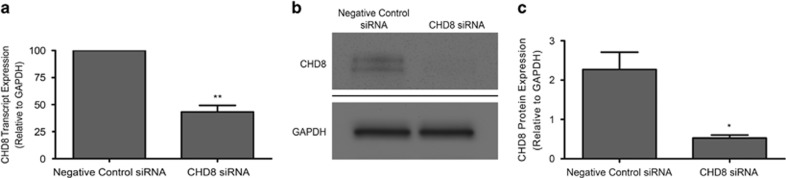
Quantitative modeling of disruptive mutations in CHD8. (**a**) Quantitative PCR showed that a 20 nM concentration of *CHD8* siRNA resulted in an ~50% reduction of *CHD8* transcript. (**b**) Representative western blot indicated decreased CHD8 protein following *CHD8* siRNA-mediated knockdown. (**c**) Quantitative analysis of western blot data shows a significant reduction in CHD8 protein following *CHD8* siRNA treatment. Error bars are s.e.m., **P<0.01, *P<0.05. CHD8, chromodomain helicase DNA-binding protein 8; siRNA, small interfering RNA.

The ASD-associated *CHD8 de novo* LoF mutations likely result in an ~50% reduction in *CHD8* transcript and CHD8 protein in the developing brains of individuals with ASD. To quantitatively model these dosage-dependent consequences of *CHD8* LoF mutations, siRNA-mediated knockdown of *CHD8* was performed in the human neural progenitor cell line, SK-N-SH. Following this, the knockdown efficiency of both *CHD8* transcript and CHD8 protein levels were confirmed via qPCR and western blotting, respectively (Figure 1).

### CHD8 insufficiency results in genome-wide transcriptional changes

**Figure 2:**
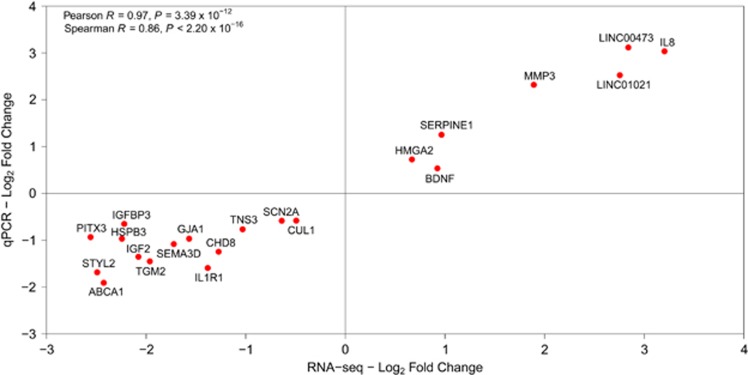
qPCR validation of differentially expressed genes identified by RNA-Seq. Fifteen genes excluding *CHD8* were assayed via qPCR to establish the validity of the differential expression analysis based on the obtained RNA-seq data. qPCR and RNA-seq values showed a statistically significant correlation with both Pearson and Spearman methods. qPCR, quantitative PCR.

As transcriptional changes due to CHD8 insufficiency may be able to inform the mechanisms by which a lack of *CHD8* influences ASD pathogenesis, we performed genome-wide transcriptome profiling of *CHD8*-depleted human neuronal progenitor cells, SK-N-SH, via RNA-seq. Differential gene expression analysis using the UCSC Hg19 genome annotation revealed 1715 differentially expressed genes (Supplementary Tables 2 and 3), with a majority of protein-coding genes downregulated (~63%). Subsequent qPCR validation was performed for genes categorized as either having large fold changes in expression or possessing functions related to neurodevelopmental processes identified by GO analysis. Comparison of gene expression levels measured by RNA-Seq and qPCR indicated a highly significant Pearson correlation coefficient (Figure 2), validating the magnitude of gene expression changes identified by RNA-seq. Both protein-coding and ncRNAs with altered expression are distributed across the genome in a pattern consistent with length of the chromosome; there does not appear to be any clustering of the genes with altered expression (Supplementary Figures 1 and 2).

### CHD8 influences the expression of ncRNA

NcRNAs represent a relatively unexplored source of functional transcripts that may contribute to neurodevelopmental phenotypes.^26^ Using the ENSEMBL GrCH37, release 75, gene annotation, we identified 657 differentially expressed genes and found that ~12.5% of these were ncRNAs (Table 1, Supplementary Tables 4 and 5). The identified ncRNA account for 40% of the top 10 differentially expressed genes (Table 1). In contrast to protein-coding genes, the majority of ncRNAs are upregulated (~60%) instead of downregulated following *CHD8* knockdown.

### Loss of functional CHD8 affects numerous ASD susceptibility genes

Comparing the list of differentially expressed genes to those genes contained in the SFARI AutDB gene database,^24^ there is an overlap of 65 genes excluding *CHD8* (Supplementary Table 6). Of the 631 genes in the AutDB database, 409 were expressed in SK-N-SH cells. Among the 1715 genes differentially expressed following *CHD8* knockdown in SK-N-SH cells, 65 were overlapping (Supplementary Table 7). Although the entire list of differentially expressed genes does not reach statistical significance for enrichment (*P*=0.059, OR (odds ratio)=1.307, Fisher's exact test), considering only those genes that are downregulated significantly strengthens this enrichment (*P*=4.793 × 10^−3^, OR=1.59). To focus on genes with substantial evidence for a role in ASD, a restricted list of 250 genes (159 of which are expressed in SK-N-SH cells) was curated from the SFARI database taking into account SFARI gene rankings as previously described.^25^ Following this narrowing, the list of differentially expressed genes due to knockdown of *CHD8* contained a total of 27 overlapping genes excluding *CHD8* (Table 2), which did not reach statistical significance for enrichment (*P*=0.153, OR=1.378). Again, considering only those genes that are downregulated significantly strengthened the enrichment (*P*=0.038, OR=1.673). In comparing all genes from the SFARI database and the narrow list of ASD genes, upregulated genes fail to show significant enrichment in both the cases. These results suggest no advantage of narrowing the list of genes by evidence-based scoring. These results further suggest that CHD8 insufficiency may regulate a network of genes that contribute to ASD risk. Although previously identified ASD candidate genes are enriched among the 1715 differentially expressed genes following *CHD8* knockdown, the magnitude of gene expression changes is similar between ASD candidate genes and genes not previously implicated in ASD. These results suggest that, in addition to altering expression of genes implicated in ASD, *CHD8* knockdown also impacts expression of genes that are either unrelated to ASD or not yet identified as contributors to ASD risk.

### Transcriptional consequences of CHD8 insufficiency are enriched in processes essential for neuronal development

**Figure 3:**
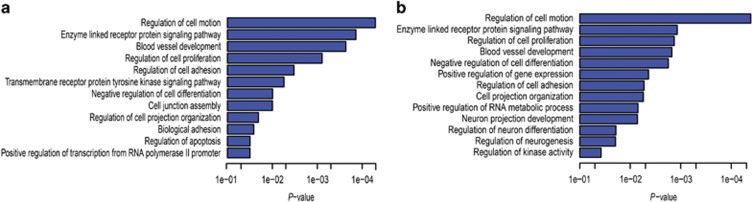
Representative enrichment of gene ontology among differentially expressed genes. (**a**) Gene ontology (GO) enrichment analysis on all differentially expressed genes due to knockdown of *CHD8*. (**b**) GO enrichment analysis on genes downregulated in response to knockdown of *CHD8*. Upregulated genes subjected to GO analysis indicated enrichment of mitochondrial membrane genes only (not shown). *P*-value represents Benjamini–Hotchberg correction for multiple tests. CHD8, chromodomain helicase DNA-binding protein 8.

GO analysis using the DAVID web server for all differentially expressed genes shows an enrichment of genes within GO categories describing several functions that have previously been associated with CHD8 including regulation of proliferation, apoptosis and transcription (Figure 3a, Supplementary Table 8).^11, 12, 27^ We also performed GO analysis grouping the differentially expressed genes by their direction of differential expression. Although upregulated genes show GO enrichment in mitochondrial membrane-related terms (Supplementary Table 9), GO analysis of downregulated genes shows an enrichment in several neuron-specific processes in addition to those categories observed for all differentially expressed genes. These include GO categories such as regulation of neuron differentiation, neurogenesis and neuron projection development (Figure 3b, Supplementary Table 10), all of which may be processes disrupted during ASD pathogenesis. In addition, utilizing the Uniprot tissue (UP_TISSUE) annotation database, we found the downregulated genes to be highly enriched in brain tissue (*P*=2.01 × 10^−15^, Benjamini–Hotchberg correction), whereas upregulated genes were not enriched in any specific tissue (Supplementary Table 11). Overall, these results suggest that CHD8 retains conserved functions and also has an essential role in the maintenance of tissue-specific genes involved in neuron development.

### CHD8 regulates a highly interconnected network of genes

**Figure 4:**
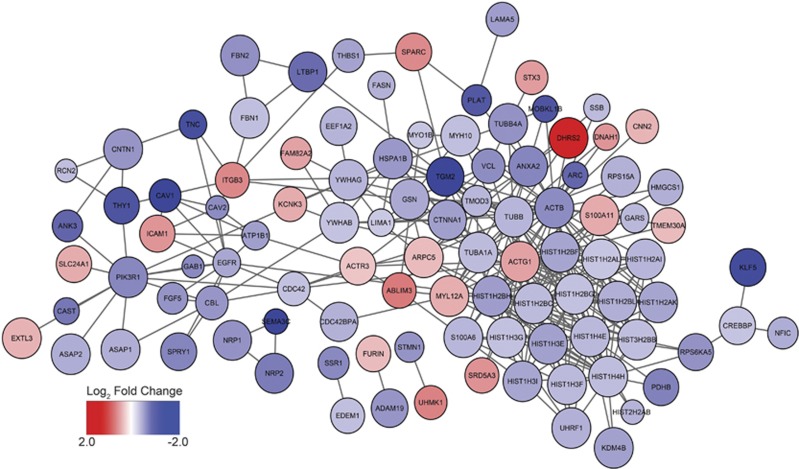
Protein–protein interaction analysis of differentially expressed genes—Disease Association Protein–Protein Link Evaluator (DAPPLE) was used to determine direct relationships among the protein products of the genes that were differentially expressed due to knockdown of *CHD8*. A subnetwork of genes with associated corrected *P*-values <0.05, indicating seed proteins that are more likely to be connected to other seed proteins within the network than by chance, was generated and visualized using Cytoscape software. Increasing significance corresponds to increasing node size. Differential expression of the associated gene product is shown with increasing densities of blue and red indicating the degree of down- and upregulation conferred by knockdown of *CHD8*, respectively. CHD8, chromodomain helicase DNA-binding protein 8.

To assess the connectivity among the differentially expressed genes following knockdown of *CHD8*, we utilized the Disease Association Protein–Protein Link Evaluator.^22^ Analysis of all differentially expressed genes shows significant enrichment in both direct and indirect connectivity (*P*=3.00 × 10^−3^ for both), identifying 2022 direct interactions (Figure 4, Supplementary Table 12). Analysis of the downregulated genes indicated a significant increase in indirect connectivity (*P*=3.00 × 10^−3^ for direct connectivity and *P*=9.99 × 10^−4^ for indirect connectivity). Although upregulated genes showed no enrichment in ASD candidate genes and weak enrichment in GO categories, Disease Association Protein–Protein Link Evaluator showed an unexpected enrichment in direct and indirect connectivity among upregulated genes (*P*=2.00 × 10^−3^ and *P*=3.20 × 10^−2^, respectively). These results suggest a network of connected genes upregulated by *CHD8*.

## Discussion

From the initial discovery through exome sequencing to confirmation by targeted sequencing of additional ASD probands, *de novo* LoF mutations in *CHD8* are highly implicated in contributing to ASD.^5, 6, 7, 8, 9^ Due to the heterogeneity of ASD, it is likely to be influenced by the combinatorial effects of hundreds of genes under abnormal regulation. Although genetic influences may differ among ASD cases, strong evidence suggests that the functional contribution of these various factors may converge on similar pathways.^4, 28^ Our data suggest that *CHD8* may be a central node in one of these pathways as CHD8 insufficiency in human neural progenitor cells results in altered expression of previously identified ASD candidate genes. Among recently identified genes disrupted in autism,^29^ *CACNA2D3* and *ETFB* were among the 1715 genes with altered expression following CHD8 knockdown, suggesting potential biological pathways that contribute to ASD.

Considering the enrichment of ASD candidate genes among the differentially expressed protein-coding genes, the ncRNA genes identified in our analysis may also serve as contributing factors to ASD phenotypes. The ncRNA represents a relatively unexplored category of genes that participate in several regulatory functions and may potentially contribute to the neurodevelopmental phenotypes observed in ASD.^26, 30, 31^ For example, the ncRNAs colorectal neoplasia differentially expressed (CRNDE) and telomerase RNA component, have both been shown to undergo differential expression during neuronal differentiation of pluripotent stem cells suggesting their participation in neurogenesis.^32, 33^ In particular, CRNDE is intimately linked with the insulin/IGF signaling pathway, as treatment with IGF1 or IGF2 downregulates nuclear-retained CRNDE transcripts.^34^ This is of importance to ASD as administration of IGF1 has been shown to rescue phenotypes associated with Rett Syndrome^35, 36^ and is now in clinical trials.^37^ Our findings of upregulation of CRNDE following decreased expression of *CHD8* suggest that this ncRNA may represent a novel therapeutic target. In addition, our data shows downregulation of the ncRNA TUG1, which has been shown to directly associate with the chromatin modifying complex, PRC2, and to be regulated by p53.^38^ This may represent a novel ncRNA target of CHD8, as CHD8 is known to interact with p53 and colocalize to promoters of p53-regulated genes.^11^

Consistent with previous studies showing the participation of CHD8 in large protein complexes involving members of the mixed lineage leukemia (MLL) family,^39^ depletion of endogenous CHD8 in neural cells results in decreased expression of the MLL genes, *MLLT1* and *MLLT3*, along with *MN1*, which associates with MLL to regulate transcription.^40^ In addition, our data show the conservation of specific changes in gene expression including genes involved in Wnt signaling (*DKK1* and *NKD2*) and the p53 target, *CDKN1A*, observed in *CHD8*-depleted HCT116 colorectal cancer cells and U2OS osteosarcoma cells, respectively.^11, 41^ Approximately 13% of differentially expressed genes in our analysis of human neural progenitor cells were shown to have CHD8 bound to their promoters in previous work characterizing the binding sites of CHD8 in human C33A cervical carcinoma cells (Supplementary Table 13).^14^ Although these data point toward the conservation of specific regulatory mechanisms involving CHD8, our analyses also suggest a tissue-specific function of CHD8 in maintaining the active transcription of genes related to neuronal development. This is illustrated by the significant enrichment of neuron-development GO categories, brain tissue specificity and the significant connectivity of genes downregulated by CHD8 insufficiency. In contrast, upregulated genes lack tissue enrichment, suggesting that they are not specific to any one particular tissue type.

Sugathan *et al.*^42^ recently described the binding sites of CHD8 and changes in transcription resulting from knockdown of *CHD8* in iPSC-derived neural progenitor cells where they found *CHD8* to be localized to 5568 promoters and 1756 differentially expressed genes, respectively. Comparing the Sugathan *et al.*^42^ list of differentially expressed genes with the list of 1715 genes observed in the current study, there is an overlap of 159 genes (9.37%, Supplementary Table 14). There were 633 (37.30%) differentially expressed genes in our data set that Sugathan *et al.*^42^ reported to have CHD8 bound in close proximity to the transcription start site (Supplementary Table 15), indicating that there is a substantial proportion of genes directly regulated by CHD8. In addition, the differentially expressed genes that are shared between our data and that of Sugathan *et al.*^42^ include *H1F0*, *TP53INP1* and *MLLT3*, suggesting conserved functions of CHD8 to participate in protein complexes containing members of the MLL family^39^ and regulate p53 signaling through interactions with Histone H1.^11^ As we also find *CHD8* knockdown results in an enrichment of differentially expressed ASD-associated genes, this illustrates the involvement of *CHD8* in regulating neurodevelopmental genes, which may differ between cell types and emphasizes the need for these transcriptional networks to be analyzed in several cellular models.

Our data indicate that a decrease of functional *CHD8* in human neural progenitor cells induces numerous transcriptional changes that potentially contribute to the pathogenesis of ASD. As the goal of this study was to uncover the consequences of CHD8 insufficiency as a whole rather than each of the particular *CHD8* LoF mutations identified in individual patients, the modeling of the exact *CHD8* LoF mutations through genome-editing techniques or induced pluripotent stem cell generation should be considered a priority. With the function of CHD8 in neurons just beginning to be explored, this work lays a foundation for further functional studies and identifies numerous molecular targets, including targetable ncRNAs, that could potentially be modulated for the purposes of therapeutic intervention.

## Figures and Tables

**Table 1:** Top 10 differentially expressed genes following knockdown of *CHD8*

*Gene*^a^	*Fold change*	*Locus*^a^	*Protein- or noncoding*	Q*-value*
IL8	7.60	4:74606222–74609433	Protein-coding	2.76E−3
LINC00473	6.53	6:165740775–166401536	Noncoding	2.03E−2
LINC00877	5.59	3:72084450–72291716	Noncoding	2.93E−2
ACPP	5.03	3:132036210–132087142	Protein-coding	1.53E−2
LINC01021	4.66	5:27472398–27496508	Noncoding	2.76E−3
HSPB3	−4.64	5:53751444–53752207	Protein-coding	1.68E−2
ABCA1	−4.89	9:107543282–107691173	Protein-coding	2.76E−3
SYTL2	−5.51	11:85405266–85522184	Protein-coding	2.76E−3
PTX3	−5.89	3:156893011–157251408	Protein-coding	2.76E−3
RP11-777B9.5	−6.81	4:78929665–78929842	Noncoding	2.76E−3

Abbreviation: CHD8, chromodomain helicase DNA-binding protein 8.

a Refers to ENSEMBL human genome GrCH37 build 37.

**Table 2:** ASD-associated genes differentially expressed following knockdown of *CHD8*

*ASD-associated gene*^a^	*Fold change*	*Locus*^b^	Q*-value*
HTR1B	4.22	6:78171947–78173120	3.54E−2
CNTNAP4	1.88	16:76311175–76593135	1.27E−2
NBEA	1.65	13:35516423–36705514	1.05E−3
CNTN4	1.63	3:2140549–3099645	1.05E−3
ITGB3	1.55	17:45331207–45390077	9.79E−3
SYNE1	1.47	6:152442818–152958534	1.05E−3
STK39	1.28	2:168810529–169104105	2.55E−2
GTF2I	−1.27	7:74072029–74175022	2.20E−2
UBE2H	−1.31	7:129470572–129592800	7.70E−3
RIMS3	−1.34	1:41086351–41131324	4.08E−3
ICA1	−1.34	7:8152814–8302242	7.65E−3
RPS6KA2	−1.37	6:166822853–167275771	1.92E−3
NRXN2	−1.38	11:64373645–64490660	1.71E−2
ADK	−1.39	10:75910942–76469061	1.71E−2
CEP41	−1.40	7:130033611–130081051	7.65E−3
HDAC4	−1.40	2:239969863–240322643	1.12E−2
NF1	−1.41	17:29421944–29704695	3.40E−3
TSPAN7	−1.45	X:38420730–38548172	2.47E−2
SCN2A	−1.56	2:166095911–166248820	1.05E−3
REEP3	−1.59	10:65281122–65384883	1.05E−3
CHRNA7	−1.63	15:32322685–32462384	1.92E−3
OXTR	−1.66	3:8792094–8811300	1.40E−2
NRP2	−1.68	2:206547223–206662857	1.05E−3
DHCR7	−1.70	11:71145456–71159477	1.05E−3
SEMA5A	−1.81	5:9035137–9546233	1.05E−3
KCNMA1	−1.83	10:78629358–79397577	1.05E−3
NPAS2	−2.45	2:101436612–101613287	1.05E−3

Abbreviations: ASD, autism spectrum disorder; CHD8, chromodomain helicase DNA-binding protein 8.

a According to genes within SFARI database possessing gene scores of ‘1–4' and ‘S'.

b Refers to UCSC Hg19.
